# Neurofeedback for Tinnitus Treatment – Review and Current Concepts

**DOI:** 10.3389/fnagi.2017.00386

**Published:** 2017-12-01

**Authors:** Dominik Güntensperger, Christian Thüring, Martin Meyer, Patrick Neff, Tobias Kleinjung

**Affiliations:** ^1^Neuroplasticity and Learning in the Healthy Aging Brain (HAB LAB), Department of Psychology, University of Zurich, Zurich, Switzerland; ^2^University Research Priority Program ‘Dynamics of Healthy Aging’, University of Zurich, Zurich, Switzerland; ^3^Department of Otorhinolaryngology, University Hospital of Zurich, Zurich, Switzerland

**Keywords:** tinnitus, phantom perception, EEG, plasticity, heterogeneity, neurofeedback, frequency bands, alpha band

## Abstract

An effective treatment to completely alleviate chronic tinnitus symptoms has not yet been discovered. However, recent developments suggest that neurofeedback (NFB), a method already popular in the treatment of other psychological and neurological disorders, may provide a suitable alternative. NFB is a non-invasive method generally based on electrophysiological recordings and visualizing of certain aspects of brain activity as positive or negative feedback that enables patients to voluntarily control their brain activity and thus triggers them to unlearn typical neural activity patterns related to tinnitus. The purpose of this review is to summarize and discuss previous findings of neurofeedback treatment studies in the field of chronic tinnitus. In doing so, also an overview about the underlying theories of tinnitus emergence is presented and results of resting-state EEG and MEG studies summarized and critically discussed. To date, neurofeedback as well as electrophysiological tinnitus studies lack general guidelines that are crucial to produce more comparable and consistent results. Even though neurofeedback has already shown promising results for chronic tinnitus treatment, further research is needed in order to develop more sophisticated protocols that are able to tackle the individual needs of tinnitus patients more specifically.

## Introduction

Subjective tinnitus has been described as the constant perception of an auditory sensation that does not correlate to any external acoustic stimulus (Stouffer and Tyler, 1990). It can be perceived as either pitch or noise-like sound and its perception may be unilateral, bilateral or spread out in the whole head (De Ridder et al., 2014b). In industrialized countries, roughly 10% of the population is affected by this stressful condition and many people suffer from sleeping or concentration problems, affected social interactions and psychological distress that can also lead to severe depression or anxiety impairments (Heller, 2003; Henry et al., 2005). The relatively large percentage of affected people, recently developed neuropsychological models, and the fact that, to date, no satisfactory potent treatment has been discovered may explain the increasing interest in tinnitus research. New findings on the pathophysiology of tinnitus have led to the development of several promising neuromodulatory techniques that have been shown to relieve symptoms of the chronic acoustic sensation and significantly increase quality of life for tinnitus sufferers (e.g., Eggermont and Roberts, 2004; Weisz et al., 2007a). One of them is neurofeedback, an already well-established form of neuropsychological treatment that recently enjoys great popularity due to its non-invasive nature, its long-lasting effects, its easy-handling and relatively low cost, as well as its rapid technological improvements. The purpose of this review is to summarize and discuss findings of neurofeedback studies for the treatment of chronic tinnitus. The focus is hereby laid on neurofeedback based on electrophysiological recordings with electroencephalography (EEG) or magnetoencephalography (MEG) but also a short summary of new innovative methods (e.g., real-time functional Magnetic Resonance Imaging, rt-fMRI) will be given. In a first step, an overview about popular models of tinnitus genesis will be provided, and studies investigating chronic tinnitus with EEG or MEG will be presented and critically discussed. Next, the development and history of neurofeedback will be briefly introduced and the different neurofeedback protocols used in tinnitus treatment summarized and evaluated. Finally, limitations of existing treatment studies will be discussed, and implications for future studies will be given.

## Tinnitus Models and Electrophysiological Studies

Tinnitus was first assumed to be solely generated in the ear or by a dysfunction of the auditory nerve (Møller, 1984; Eggermont, 1990), but the focus of attention quickly shifted to the human brain after Jastreboff (1990) proposed what is nowadays known as the *neurophysiological model of tinnitus*. Even though some form of inner ear damage indeed seems to be a necessary prerequisite, Jastreboff (1990) suggested central processes in the auditory cortex, the limbic system, and prefrontal areas to be crucial for tinnitus genesis. Later models picked up this idea and tried to specify the neuroplastic alterations emerging after auditory deafferentation. In this context, an increase in central gain in subcortical structures of the auditory pathway (Noreña, 2011), reorganization of tonotopic maps in the primary auditory cortex (Mühlnickel et al., 1998), a thalamocortical dysrhythmia (Llinás et al., 1998, 1999, 2005; Weisz et al., 2007a) and changes in neural synchrony (Noreña and Eggermont, 2003; Seki and Eggermont, 2003; Eggermont and Roberts, 2004; Weisz et al., 2005), or a failing top-down noise-canceling mechanism (Rauschecker et al., 2010, 2015) have been discussed. Furthermore, global workspace models emphasize the importance of networks beyond the auditory system (De Ridder et al., 2014b), and frameworks of filling-in missing auditory information have been suggested in a Bayesian way (De Ridder et al., 2006, 2011, 2014a) or based on predictive coding (Sedley et al., 2016).

### First Wave of Electrophysiological Studies

Apart from animal experiments, brain imaging and morphometry studies, the investigation of resting-state brain activity with electrophysiological methods, such as EEG or MEG, enjoys great popularity in tinnitus research (Adjamian, 2014). In order to pinpoint neural correlates of the ongoing tinnitus sensation, first studies compared spontaneous brain activity of tinnitus patients at rest with the one of healthy controls. In this context, most investigations focused on the analysis of neuronal oscillations separated into distinct frequency bands: delta (0.5–4 Hz), theta (4.5–8 Hz), alpha (8.5–12 Hz), beta (12.5–35 Hz), and gamma (35.5–80 Hz). Following this approach, early studies (Weisz et al., 2005, 2007b; Ashton et al., 2007; Kahlbrock and Weisz, 2008; Lorenz et al., 2009) found a relatively consistent pattern of enhanced activity in delta- and gamma frequencies, alongside with reduced amounts of alpha oscillations over temporal areas of tinnitus patients (for a review, see Schlee et al., 2008; Adjamian et al., 2009). These findings have been interpreted in the framework of the *thalamocortical dysrhythmia model* (TCD), originally proposed by Llinás et al.’s (1998, 1999, 2005) and later significantly refined by Weisz et al. (2007a) to the *synchronization by loss of inhibition modulation* (SLIM) model. Both models aim at sketching tinnitus genesis as the result of an imbalance between inhibition and excitation in thalamocortical circuits. Loss of sensory input (deafferentation) gives raise to low frequent self-oscillations of thalamic cells which activate the auditory cortex and can thus be measured as oscillations in a slow delta rhythm on the scalp. At the same time, input deprivation also leads to a downregulation of inhibitory mechanisms which is reflected in alpha desynchronization in the resting-state EEG or MEG. This decrease of inhibition is then proposed to lead to spontaneous synchronization of firing reflected in increasing activity in fast gamma oscillations. This pattern of increased resting-state delta and gamma and decreased alpha has thus been termed the *neural signature of tinnitus*, and gamma has been interpreted as the neuronal substrate of the sound percept itself.

### Limitations of the Early Studies

One of the major flaws of these early studies, however, was that they did not consider that chronic tinnitus is a very heterogeneous phenomenon and can differ substantially between individuals. It has clearly been shown that the subjective experience of the chronic sound (intensity, pitch, location) as well as the related distress and comorbid symptoms vary considerably among sufferers (Landgrebe et al., 2010; Langguth et al., 2013; Weidt et al., 2016; van den Berge et al., 2017). In addition, the underlying neuroanatomical and neurophysiological alterations may be far from homogenous in the population of tinnitus patients. Instead of comparing tinnitus patients with healthy controls, more recent studies thus focused on differences *within* the tinnitus sample with the ultimate goal of identifying distinct subtypes of tinnitus and finding different forms of treatment for each of these subtypes.

Another issue that the earlier studies had to deal with is the fact that electrophysiological methods suffer from rather poor spatial resolution. In terms of neuroscience, the *inverse problem* describes the fact that signal as measured by electrodes or magnetometers on the scalp could be generated by infinite combinations of neuronal sources (Scherg and Berg, 1991). The described pattern of tinnitus-specific oscillations found in the earlier studies, even though measured over temporal areas, could therefore have been generated in (or significantly altered by) cell assemblies outside of the primary auditory cortex. Different *source estimation algorithms* have been developed in the recent past to solve this inverse problem as well as possible by applying different *a priori* assumptions. With these algorithms the source of a measured signal can be estimated and spatial resolution of resting-state EEG and MEG measurements significantly increased (Michel et al., 2004). Standardized Low Resolution Electromagnetic Tomography (sLORETA) (Pascual-Marqui, 2002) or beamformer algorithms (van Veen et al., 1997; Hillebrand et al., 2005; Grosse-Wentrup et al., 2009) are examples of fairly precise and therefore relatively popular source estimation techniques.

The new focus on differences within the tinnitus population and the improvements in electrophysiological analysis methods have led to a veritable boom of resting-state tinnitus studies. Some investigations have confirmed the neuronal tinnitus code and auditory gamma as its major brain correlate by applying sLORETA (van der Loo et al., 2009; Moazami-Goudarzi et al., 2010; Vanneste et al., 2011a) or beamformer (Ortmann et al., 2011) source estimations to the measured signal, reporting correlations between tinnitus loudness and auditory gamma (van der Loo et al., 2009) or by performing intervention studies with acoustic coordinated reset (Tass et al., 2012; Adamchic et al., 2014a,b, 2017). Schlee et al. (2014), on the other hand, found decreased power (and variability) only for the lower (8–10 Hz) but not for the upper alpha band (10–12 Hz) and other studies failed completely to find the expected pattern in the auditory areas (Vanneste et al., 2011b, 2012; Song et al., 2013; Meyer et al., 2014; Zobay and Adjamian, 2015). Furthermore, two studies (Sedley et al., 2012; Sedley and Cunningham, 2013) discussed the possibility that auditory gamma oscillations could emerge as an attempt of the brain to suppress the tinnitus percept rather than causing it.

### Tinnitus Network(s) and Areas Beyond the Auditory Cortex

In neuroscience, the gamma frequency range has also been debated as a binding medium connecting activity of various circuits to form a unified percept (Singer, 1993). Already Schlee et al. (2009) reported gamma-related abnormalities in a network with core regions in prefrontal, orbitofrontal, and parieto-occipital areas. Later the different parallel networks that may differentially contribute to the various tinnitus symptoms were described in more detail (De Ridder et al., 2011, 2014b; Vanneste and De Ridder, 2012). A *tinnitus core network* was proposed to generate the sound *per se* and code its intensity and location (holocranial, uni- or bilateral). Other networks were introduced as modulating the sound type (sine wave tone, hissing, ringing) as well as aversive states and feelings (e.g., distress or mood) of tinnitus (De Ridder et al., 2014b). An increased and persisting amount of gamma oscillations and coupling with slow-waves could thus suggest that activity of these widely-distributed brain networks is constantly *bound together* (synchronized), and a unified tinnitus percept is formed with its very own characteristics for each individual coded in the relevant sub-networks. In order to capture the tinnitus phenomenon in its entirety, areas outside of the central auditory regions therefore have to be considered. Furthermore, the specificity of the measured EEG-patterns has to be carefully validated as related disorders might produce similar findings (e.g., Joos et al., 2012; Meyer et al., 2017). These considerations are also relevant with regard to the development of neurofeedback protocols.

Apart from investigations comparing brain networks of tinnitus patients and healthy controls based on analyses with graph theory or machine learning algorithms (Mohan et al., 2016a,b, 2017a,b), a multitude of recent electrophysiological studies attempt to find specific correlates in neural networks for the different aspects of tinnitus (Adjamian, 2014; De Ridder et al., 2015; Eggermont, 2015; Elgoyhen et al., 2015). These studies mainly investigated tinnitus-related distress or loudness, but also covered tinnitus type, pitch, location/laterality, duration, age of onset, day-time awareness, or related problems such as hearing loss, hyperacusis, depression, or general quality of life (a detailed summary is provided in the Supplementary Materials). The most consistent findings are reported for tinnitus-related distress, which seems to be represented in a network ranging from structures of the limbic system (e.g., anterior cingulate cortex and amygdala) to prefrontal areas (e.g., dorsolateral prefrontal cortex), and also includes the insula. Altogether, however, the results of these studies are rather heterogeneous, and attempts of replication are scarce and partly fail to confirm previous findings (Pierzycki et al., 2015; Meyer et al., 2017). This can partially be explained by different EEG or MEG hardware used for resting-state recordings, different paradigms during the measurement [e.g., length of measurement, operationalization of tinnitus symptoms, or condition of resting-state (eyes open/closed) used for the analysis], different source estimation algorithms and data analysis procedures. To resolve this issue, scholars of the European research network *TINNET*^1^ are channeling their efforts to establishing general guidelines for (electrophysiological) tinnitus studies and collecting comparable data in a large database^2^. In order to tackle the problem of tinnitus heterogeneity, it is thus of utmost importance that future studies take these guidelines into consideration, report also null- or conflicting results and further also extend their focus to replicating previous findings.

## Neurofeedback

Applying neurophysiological methods, neurofeedback is a non-invasive neuromodulation technique which records a subject’s neuronal activity, extracts relevant aspects of brain processes by means of real time signal processing and returns feedback to the subject as visual or auditory stimuli. The aim of neurofeedback is to change behavioral traits or medical conditions associated with altered neural activity as demonstrated for chronic tinnitus in the previous section. This is generally done by means of operant conditioning (i.e., rewarding of wanted, inhibiting of unwanted changes) whereby the subjects learn to voluntarily change their own brain activity in the desired direction.

### A Brief History of Neurofeedback

In the early 1930’s and 1940’s, human studies already suggested the capability of the central nervous system to alter neural activity patterns by means of conditioning methods (Loomis et al., 1936; Jasper and Shagass, 1941). Later, Wyrwicka and Sterman (1968) were able to train cats to change their brain activity in a specific direction, and, shortly after that, the first study with human subjects in this context was published (Sterman and Friar, 1972). In the following years, neurofeedback was intensively tested and showed promising results mainly in treatment studies with epilepsy and attention deficit hyperactivity disorder (ADHD) (Lubar and Bahler, 1976; Lubar and Lubar, 1984). For ADHD, neurofeedback already found acceptance as alternative to established medication based treatment, due to its non-invasive character, the almost complete absence of any side-effects and high self-efficacy experienced by the subjects (Lubar et al., 1995; Lévesque et al., 2006; Arns et al., 2009; Gevensleben et al., 2009; Strehl et al., 2017). Apart from that, effectiveness and feasibility of neurofeedback are more and more investigated in the context of many other psychological disorders and neurological conditions ranging from the treatment of depression (Kelley et al., 2017), anxiety (Mennella et al., 2017), or autism (Datko et al., 2017) to stroke patients (Kober et al., 2017) and prevention of Alzheimer’s disease (Jiang et al., 2017). Today, quality control is an important aspect in the neurofeedback field. The Biofeedback Certification International Alliance (BCIA)^3^ certifies bio- and neurofeedback practitioners who meet certain requirements and the Association for Applied Psychophysiology and Biofeedback (AAPB)^4^ recently released the 3rd edition of *Evidence-Based Practice in Biofeedback and Neurofeedback*, a document that summarizes treatment efficacy for various disorders (Tan et al., 2016).

### Common Neurofeedback Paradigms

Neurofeedback training of classical definitions of distinct frequency bands (i.e., delta, theta, alpha, beta, and gamma) are the most commonly used protocols in the current literature. The main field of frequency band neurofeedback is the treatment of ADHD, where often a combination of different frequencies is trained (Lofthouse et al., 2012). However, classic frequency band training has also been adapted for other disorders, most prominently anxiety or affective problems (Hammond, 2005). Importantly, neurofeedback training based on this paradigm ultimately depends on findings of fundamental research about disorder-specific neural alterations and can even be used to confirm or disprove these findings.

Sensorimotor rhythms (SMR) are defined as EEG oscillations in the lower beta range (12 – 20 Hz). They are generally measured over the sensorimotor cortex and proposed to originate from the ventrobasal nucleus in the thalamus (Howe and Sterman, 1972, 1973). Neurofeedback training based on SMR mainly found application in the treatment of epilepsy (Sterman and Egner, 2006) or ADHD (Monastra et al., 2002; Fuchs et al., 2003).

Slow cortical potentials (SCP’s) describe very slow oscillations in a range of 0.3–1.5 Hz. They describe slow, discrete, and continuous shifts (up to seconds) of the overall cortical distribution of electrical activity representing increased or decreased excitability of underlying neuronal structures. SCP’s are usually recorded with a single electrode in a central position (Cz) and are proposed to reflect cognitive or motor preparation (Hammond, 2011). Initially, SCP training was exclusively applied in trials with patients suffering from epilepsy (Rockstroh et al., 1993) but later also found application in the treatment of ADHD (Strehl et al., 2017).

Infra-low neurofeedback (ILN) relies on training of even slower brain oscillations, ranging from 0.001 to 1.5 Hz (Vanhatalo et al., 2004). Infra-low oscillations were shown to correlate with other frequency bands as well (Monastra et al., 2002). There is an overlap with SCP-based neurofeedback, which mainly differs in the recording of SCP’s with a single central electrode and thus a training of a more summarized potential over the whole head. Positive effects of ILN on different neurological conditions were reported in case reports (Legarda et al., 2011).

In *z*-score neurofeedback, the training protocol for an individual patient is based on previous recordings of EEG data and comparison to a healthy age-matched normative database (Thatcher, 2010). During the neurofeedback training, patients try to normalize their EEG patterns and minimize deviations from this control group. This NFB alternative is a rather data-driven technique, and some studies report successful treatment of various disorders (e.g., schizophrenia, addiction, ADHD, or personality, anxiety, and affective disorders) with *z*-score neurofeedback (Surmeli and Ertem, 2009; Surmeli et al., 2012; Simkin et al., 2014).

Functional magnetic resonance imaging (fMRI) was introduced to the field of neurofeedback to obtain a better spatial resolution. Real-time acquisition of blood oxygenation level dependent (BOLD) signals demonstrates increased neural activity according to higher oxygen supply to active neurons (Ogawa et al., 1990). Although newer to the field, a large quantity of clinical treatment studies already focused on the use of real-time fMRI neurofeedback (Sulzer et al., 2013). The higher spatial resolution of fMRI neurofeedback, however, does not come without limitations. Increased blood oxygenation can be measured only after a delay of several seconds and is an indirect correlate of underlying neuronal processes. Compared to electrophysiological methods, the temporal resolution of fMRI is thus rather poor, and fast fluctuations cannot be captured accordingly and used for the feedback. Additionally, it is questionable if an MRI-scanner is a favorable setting to perform neurofeedback because of the limited space and the loud constant background noise. For tinnitus patients, this is a huge drawback, in particular in those individuals suffering from additional hyperacusis.

To address the poor spatial resolution of single- or multi-electrode EEG and MEG recordings, neurofeedback techniques have also been combined with source estimation algorithms. Congedo et al. (2004) introduced the first tomographic neurofeedback protocol based on the inverse solution technique *LORETA* (Pascual-Marqui et al., 1994). This approach has subsequently been intensely tested mainly in the context of ADHD treatment (Cannon et al., 2006, 2007, 2009, 2014; Koberda et al., 2012, 2013) and has recently been further refined (Congedo, 2006; Pllana and Bauer, 2011; Kopřivová et al., 2013; Bauer and Pllana, 2014; White et al., 2014).

### Neurofeedback and Tinnitus: Existing Studies

Presently, only a handful of studies investigated the efficacy of neurofeedback in the treatment of chronic tinnitus according to standard searching tools such as *PubMed*^5^. An overview is provided in **Table 1**.

**Table 1:** Summary of studies investigating neurofeedback for treatment of tinnitus.

Authors	Tinnitus patients	Neurofeedback	Electrodes/Sources	Feedback	Behavioral findings	Neuronal findings
Crocetti et al., 2011	*N* = 15	α↑ δ↓ 12 sessions	F3, F4, Fc1, Fc2	Plane moving up and down (with audio-visual reinforcement)	Distress ↓ Loudness ↓	α/δ-ratio ↑ (not all participants were able to manipulate α and δ successfully)
Dohrmann et al., 2007a,b	Group 1 (*n* = 11) Group 2 (*n* = 5) Group 3 (*n* = 5) Controls (*n* = 27)	Group 1: α↑ δ↓ Group 2: α↑ Group 3: δ↓ Control: FDT 10 sessions	F3, F4, Fc1, Fc2	Fish moving up and down	All groups: Distress ↓ Loudness↓ Group 1: strongest relief Controls: no reduction	All groups: α↑ and δ↓ Correlation with decrease in loudness
Gosepath et al., 2001	*N* = 40 Controls (*n* = 15)	α↑ β↓ 15 sessions	P4	Auditory and visual (not further explained)	Distress ↓	Group 1 (*n* = 24): α↑ Group 2 (*n* = 16): β↓ Controls: no effect
Hartmann et al., 2013	*N* = 8 Controls (*n* = 9)	α↑ 10 sessions Controls: rTMS	Source space projection on two temporal sources	Smiley	Distress ↓ Controls: no reductions	α↑ estimated over r PAC
Schenk et al., 2005	Group 1 (*n* = 23) Group 2 (*n* = 13)	Group 1: α↑ Group 2: β↓ Group 3: α↑ β↓	Group 1: P4 Group 2: C3	Floating ball and melody	Distress ↓	Both groups: α↑
Vanneste et al., 2016	Group 1 (*n* = 23) Controls 1 (*n* = 17) Controls 2 (*n* = 22)	Group 1: α↑ β↓ γ↓ 15 sessions Controls 1: α↑ β↓ γ↓ Controls 2: passive	sLORETA Group 1: PCC Controls 1: LG	Green bar moving up and down	Group 1: distress ↓ Controls: no reduction	No alterations in target areas for α, β and γ Changes in functional and effectivity connectivity
Weiler et al., 2002	*N* = 1	α↑ β↑ δ↑ 𝜃↑	19 electrodes	Varying	Depression ↓ Anxiety ↓ Tinnitus ↓	No analysis

*↑, increase; ↓, decrease; r PAC, right primary auditory cortex; PCC, posterior cingulate cortex; LG, lingual gyrus.*

In the first study in this context published by Gosepath et al. (2001), 40 patients suffering from chronic tinnitus and 15 control subjects underwent neurofeedback training. The training protocol included alpha training (8–13 Hz) alongside with a reduction of beta oscillations (14–30 Hz). While one group of patients (*n* = 24) was able to only increase their alpha activity, the effects of the other group (*n* = 16) were limited to the decrease of beta oscillations. All patients, however, reported to be less disturbed by their tinnitus after the training, indicated by significant decrement in scores of the tinnitus questionnaire (TQ) (Goebel and Hiller, 1994). Control subjects underwent identical trainings but without real-time feedback and did thus not show any changes in alpha or beta activity. Schenk et al. (2005) aimed at replicating the findings from Gosepath et al. (2001) with the aforementioned protocol. Before assigning them to different study groups, participants underwent baseline EEG-recordings at rest and during a stress test. Participants (*n* = 40) were assigned to three different groups according to their results. Twenty-three subjects showing decreased alpha activity under stress were allocated to a first group and set to train alpha activity (8–13 Hz) in the subsequent neurofeedback training. The second group consisted of 13 patients with increased beta activity in the stress condition and their treatment protocol thus aimed at the decreasing of beta oscillations (14–30 Hz). Four patients could not be assigned to either of the aforementioned groups according to their spontaneous brain activity and hence were allocated in a third group that had to increase alpha and decrease beta activity simultaneously. Subjects of the first group were able to increase their alpha activity, whereas subjects of the second group failed to significantly decrease their amount of beta oscillations. Surprisingly, also subjects of the second group showed increases in alpha activity even though it was not intended with the feedback. Reduced subjective tinnitus distress in terms of a reduction of TQ scores was reported for both groups. The third group was excluded from data analysis due to its small size.

A third rather explorative study shall briefly be mentioned. In a case report, Weiler et al. (2002) used *z*-score neurofeedback for one patient with bilateral tinnitus. The feedback protocol was based on EEG recordings prior to the training where decreased delta, theta, alpha and beta activities compared to 20 control subjects had been observed. The results indicated a normalization of depressive and anxiety symptoms and the patient reported that tinnitus was only occasionally present. However, no comparisons of pre–post changes in EEG patterns have been drawn in this study.

Even though these three first attempts to treat tinnitus with neurofeedback seemed to be promising, they should not be over-interpreted. First, the training-protocols were chosen rather arbitrarily and not based on previous findings of tinnitus-specific neural abnormalities. Moreover, the fact that patients of all groups reported significant improvements in tinnitus-related distress, regardless of their actual alterations of neural activity, speaks in favor of unspecific effects of the neurofeedback training. Especially the unintended increase of alpha activity in the second group of the study by Schenk et al. (2005) suggests that a general relaxation effect might have had a bigger impact than the actual neurofeedback protocol. In general, these first three studies rather aimed at helping their patients relax and reduce their general level of stress, and it is thus not surprising that reduced distress was reported after the training. However, since knowledge about the origins of tinnitus was still rare at this time, these studies can clearly be seen as pioneering works in the treatment of tinnitus with neurofeedback.

The TCD-model by Llinás et al. (1999, 2005) and the proposition of the *neural signature of tinnitus* (Weisz et al., 2007a) gave rise to new and potentially more appropriate neurofeedback protocols. Dohrmann et al. (2007a,b) developed their neurofeedback protocols by reference to these findings and aimed at an increasing of alpha and a decreasing of delta activity. Twenty-one patients suffering from chronic tinnitus were included into their study and further assigned to three different treatment groups (see **Table 1**). For the neurofeedback application 4 fronto-central electrodes (F3, F4, Fc1, and Fc2) were chosen because the recorded signal is most likely generated in the auditory cortex according to the authors. For a forth group of tinnitus patients (*n* = 27) frequency discrimination training (FDT) was applied aiming at a change of hearing-loss induced cortical map reorganization. Data analysis showed a significantly increased ratio between alpha and delta activity for the three neurofeedback groups suggesting an increase of alpha alongside with a decrease of delta over temporal auditory regions. These alterations were also correlated with a significant decline of tinnitus loudness for tinnitus patients. Subjects who were able to modify both bands simultaneously in the desired way showed the strongest relief from tinnitus compared to other groups (i.e., subgroups of patients with only alpha-, only delta-, or no change). Furthermore, the training generally resulted in a reduction of tinnitus related distress that was still notable even 6 months after the termination of the training. No statistically meaningful effects regarding tinnitus loudness or distress were found in the FDT group. In order to replicate these findings, Crocetti et al. (2011) conducted a study with 15 normal hearing tinnitus patients and tried to train them in decreasing delta and increasing alpha frequency bands. Even though no significant differences between pre- and post-training EEG patterns have been found, the results suggested an obvious trend toward an increasing alpha/delta ratio. In addition, scores evaluated with the Tinnitus Handicap Questionnaire (THI) (Newman et al., 1996) indicated significant improvements, which were maintained after the end of the training period.

All in all, these two studies suggested the protocol of upregulating alpha and downregulating delta to be a highly promising approach in tinnitus treatment. However, the surface-based nature of the neurofeedback application by simply using four electrodes on the scalp could not ensure that the brain activity used for the feedback indeed originated in the auditory areas. To address this problem, Hartmann et al. (2013) used a 32-channel EEG system and projected the recorded activity on the surface to eight regional dipole-sources, of which two were situated in the temporal cortex. Eight subjects of this investigation received neurofeedback treatment to train an increase of alpha power and nine subjects were treated with repetitive transcranial magnetic stimulation (rTMS). With the completion of the training, only patients of the neurofeedback group showed improved tinnitus distress scores. In comparison to the control group with rTMS treatment, they achieved significantly ameliorated scores in the TQ. Additionally, a comparison of MEG resting-state activity before and after treatment combined with spatial filtering based on a LCMV beamformer algorithm (van Veen et al., 1997) revealed a significant increase of alpha activity over the right primary auditory cortex. According to Hartmann et al. (2013) this proves that alpha activity can be systematically altered in the primary auditory cortex which helps restore the disturbed excitatory–inhibitory balance of tinnitus patients.

Finally, two recently published neurofeedback studies shall be mentioned. Milner et al. (2015) used SCP neurofeedback training in a case report and could show decreased tinnitus pitch and loudness as well as a reduction of delta and theta frequencies over left hemispheric fronto-temporal and temporo-occipital electrodes which they interpret as a normalization of tinnitus-specific activity. Vanneste et al. (2016) applied neurofeedback combined with sLORETA source estimation to a group of 58 tinnitus patients. A first group (*n* = 23) of this study received alpha-up training, and beta- and gamma-down training whereby the feedback was limited on the activity that was estimated to originate over the posterior cingulate cortex (PCC). A second group of 17 tinnitus patients received the same training but for activity over the lingual gyrus and a third group (*n* = 18) did not receive any treatment at all. Decreased tinnitus distress was only found for the PCC-group but no significant changes in any frequency bands were found in the trained areas. However, decreased cross-frequency coupling (i.e., alpha to beta and alpha to gamma power nesting) in the PCC and changes in functional and effective connectivity between PCC and different areas of the distress network suggest a specific effect of this training.

Finally, even though this review mainly focuses on neurofeedback based on electrophysiological recordings, it shall be noted that also real-time fMRI protocols are currently being developed and tested for tinnitus treatment with promising results (Haller et al., 2010, 2013; Emmert et al., 2017). In their investigations, the auditory cortex of tinnitus patients is first precisely localized thanks to the good spatial resolution of fMRI, and, subsequently, neurofeedback training aiming at reducing auditory BOLD activity provided. Even though this protocol leads to the intended neuronal alterations, no significant effects on tinnitus symptoms have been reported (Emmert et al., 2017).

### Limitations of Neurofeedback Training Studies

Currently, the AAPB rates the efficacy of chronic tinnitus treatment with neurofeedback as *possibly efficacious* (level 2) (Tan et al., 2016). Although various neurofeedback training protocols showed promising results in treatment of several neurological disorders, there still remain limitations and open issues which need to be addressed. In particular, EEG- and MEG-based neurofeedback studies are often criticized about the low spatial resolution of electrophysiological recordings. Despite more refined source estimation algorithms, an uncertainty about the precision of the estimation remains, which is especially important when changes in frequency bands are considered as primary outcome measures. Studies that are able to verify specific effects in the brain areas of interest are still scarce and successful improvements of certain symptoms are thus often criticized to be the mere result of unspecific placebo effects (Thibault et al., 2016, 2017). Expectations of researcher and participant, the treatment condition in general (e.g., taking time off from a busy work schedule) and interactions with the practitioner (such as, the simple meeting with a clinician) can contribute greatly to the improvement of psychological symptoms. This problem is especially predominant in the context of chronic tinnitus therapy where most participants turn to neurofeedback hopefully after repeatedly being told by their doctors that nothing can be done to treat tinnitus and having undergone a wide variety of (sometimes rather questionable) treatments on their own.

One way to resolve this issue is to improve study designs and conduct double-blind trials with control groups using a form of sham neurofeedback. In this context, Thibault et al. (2016) suggest the use of prerecorded feedback of other participants, feedback of another disease-unrelated brain area, or inverse feedback protocols that reward unwanted and inhibit wanted changes of brain activity. The use of sham-control is, however, difficult to establish in clinical neurofeedback trials because of several reasons. First, participation in neurofeedback treatment studies requires considerable investments in time and energy on the part of participants as they generally have to attend multiple training sessions over the course of several weeks. Furthermore, in sham-controlled clinical studies, participants always enter a trial with some form of expectation and hope to be part of the treatment group. Absent success after the first training sessions may lead to a misleading belief that they instead have been assigned to the control group which negatively affects their motivation and further success in the training process (Strehl et al., 2017). These drawbacks of placebo-controlled trials have to be considered and alleviated with appropriate designs, such as a cross-over approach where one group of participants receives sham training first while the other starts with verum treatment. In a second step the protocols are swapped so that both groups undergo sham- as well as verum-neurofeedback. In this context several authors point to the importance of a systematic investigation of non-specific factors in neurofeedback studies (Friedrich et al., 2014; Sitaram et al., 2017; Thibault et al., 2017). Appropriate knowledge about the factors favoring and the ones hindering success in neurofeedback treatment can indeed lead to a better understanding of the actual mode of action of neurofeedback as well as help improve the treatment setting in order to optimize therapy outcomes for patients.

A major flaw of previous neurofeedback studies is that most of them settle for reporting positive effects of their trained protocol. It is known, however, that there is a wide variability among the efficacy of neurofeedback treatment for different subjects. While some are able to successfully self-regulate their neural activity in the desired way and show improvements of corresponding symptoms (responders), others fail to do so (non-responders) (Friedrich et al., 2014). This issue was described as *neurofeedback inefficacy* by Alkoby et al. (2017) who provide a thorough review about this currently existing topic. In their publication, they chose 20 papers published after 2010 at random and found that only two of them reported the actual number of responders and non-responders in their studies. This, of course, hampers a proper evaluation of the feasibility of a given neurofeedback protocol for the treatment of a certain disorder. For one thing, positive effects of the training might be concealed or confounded by the negative results of non-responders in the clinical trial. Furthermore, information provided about responder and non-responder groups helps define and analyze factors for success or failure of the protocol. That is, by means of a thorough investigation of the attributes of responders and non-responders, predictors for (un-) successful neurofeedback can be identified, which can be used to improve training protocols for future patients.

Another issue in this context is the high heterogeneity among outcome measures and definitions to appropriately measure success or failure used in previous neurofeedback studies. On the one hand, it can be useful to use a wide variety of outcome measures in a clinical study in order to account for changes which might not be anticipated in the first place. For instance, it can be important to measure the general level of stress of tinnitus patients as the positive effects of neurofeedback could also be explained by a decrease of the general stress condition of the patient. However, guidelines need to be established which suggest the use of certain questionnaires or tests for a given field of interest to which scholars can relate when planning an investigation [substantial work in the tinnitus field is currently being done by Hall et al. (2016) in this context]. This will limit the amount of different outcome measures in clinical trials, promote the use of well-established and validated questionnaires, and foster direct comparability between findings of different investigations. Additionally, guidelines in the context of neurofeedback treatment need to answer the question as to what can be regarded as successful or unsuccessful training and how to distinguish responders from non-responders. Is it already sufficient that a given symptom simply changes over the course of a training in a positive way or does it have to improve by a certain amount (e.g., an increase by certain points in a questionnaire score)? What, on the other hand, needs to happen to and in between brain circuits? How and how much does neural activity have to be altered by the neurofeedback treatment so that an individual can be labeled as a responder? Even though some publications already tried to postulate criteria or guidelines (Gruzelier, 2014; Rogala et al., 2016; Enriquez-Geppert et al., 2017), many open issues remain in this regard.

## Conclusion

In this review, we summarized and discussed the current state of electrophysiological brain research in the field of chronic tinnitus as well as recent advances of neurofeedback treatment. Up to date, only a handful of studies exist that investigated feasibility of neurofeedback protocols for chronic tinnitus patients. While the first studies in this context rather focused on creating a general state of relaxation for the subject, later trials considered tinnitus-specific alterations in brain activity based on comparisons of EEG or MEG resting-state recordings between tinnitus patients and healthy controls. The main region of interest in these studies was the auditory cortex, and fairly good results have been achieved following this approach. With the newer developments in tinnitus research and the numerous investigations dealing with differences within the tinnitus population, which take into account the substantial amount of heterogeneity amongst tinnitus sufferers, also other potential tinnitus-related brain areas can be targeted in future neurofeedback studies. A good example in this regard is the recent publication by Vanneste et al. (2016) where the posterior cingulate cortex as part of the tinnitus distress network has been targeted. Furthermore, this investigation is the only neurofeedback study in the context of chronic tinnitus treatment to date that included a control group with training of a tinnitus-irrelevant brain area in its design.

To sum up, even though often criticized in the recent past, results of current studies suggest that neurofeedback seems to be a promising method for efficient tinnitus treatment and may enjoy great popularity in the future. The ultimate goal may be to develop different neurofeedback alternatives for a given subgroup of tinnitus sufferers or even establish neurofeedback on an individualized basis for each patient. In this context, multi-location and multi-frequency neurofeedback protocols with adequate source estimation algorithms, which are able to train multiple brain networks in power and maybe even connectivity changes simultaneously, can be seen as the gold standard for future neurofeedback protocols. At the moment, however, there still exist several challenges that need to be overcome. A general issue are technological aspects of electrophysiological measurements (e.g., the limited spatial precision of resting-state EEG recordings) and neurofeedback applications (e.g., the implementation of connectivity-based neurofeedback protocols) that need to be improved. Regarding the treatment of chronic tinnitus in particular, results of existing fundamental studies are still too heterogeneous in order to suffice for the development of more sophisticated neurofeedback protocols. One possibility to resolve this latter issue is by means of the establishment of general guidelines about adequate symptom assessment, measurement paradigms, and analysis methods. In this way, more coherent and comparable results should be published in order to lead to a better understanding of tinnitus heterogeneity and its underlying alterations in brain networks that could be tackled by future neurofeedback protocols. Additionally, this urgent need for guidelines has been shown to be an open issue in the field of clinical neurofeedback research in general. Clarity is needed about how to separate responders from non-responders, and which outcome domains and measurements are best suited to do so. Furthermore, also non-specific effects of the training have to be taken into account and systematic investigations about the most (or least) favorable neurofeedback settings and treatment conditions are needed.

## Author Contributions

Each author has provided substantial contributions to warrant authorship. Contributions are as follows: DG and CT equally contributed to the conception, draft and revision of the paper and are sharing first-authorship. MM, PN, and TK contributed to conception, critically revising and final approval of the manuscript.

## Conflict of Interest Statement

The authors declare that the research was conducted in the absence of any commercial or financial relationships that could be construed as a potential conflict of interest. The handling Editor declared a past co-authorship with the authors MM and TK.

## Footnotes

1 http://tinnet.tinnitusresearch.net/

2 https://www.tinnitus-database.de/

3 http://www.bcia.org

4 https://www.aapb.org

5 https://www.ncbi.nlm.nih.gov/pubmed/
