# Supplementary material for: Neurofeedback for Tinnitus Treatment – Review and Current Concepts
Source: Front Aging Neurosci. 2017 Dec 1;9:386. doi: 10.3389/fnagi.2017.00386 (PMC5717031; doi:10.3389/fnagi.2017.00386)
Supplement: Supplementary file 1 [file Table_1.pdf]

Supplementary Material

Neurofeedback for Tinnitus Treatment – Review and Current Concepts

Dominik Güntensperger, Christian Thüring, Martin Meyer, Patrick Neff, Tobias Kleinjung*

*** Correspondence:** Tobias Kleinjung: tobias.kleinjung@usz.ch

Table 1

Summary of recent electrophysiological studies investigating chronic tinnitus

Reference	Study Design	Source Estimation, Connectivity	Feature (Measurement)	Analysis	Findings
Adamchic et al. (2014a)	Intervention (ACR)	BESA (source montage)<br>Phase-amplitude CFC	Pitch	Responders with pitch change (Vs. without)	↓ ACC (θ phase) ↔ DLPFC (γ amplitude)<br>↓ ACC (θ phase) ↔ AC (γ amplitude)
Adamchic et al. (2014b)	Intervention (ACR)	BESA (source montage)<br>sLORETA	Distress (THI, VAS)	Post- Vs. Pre-treatment	↓ distress<br>↑ PAC (α) ↓ PAC (β,γ,δ,θ)
Adamchic et al. (2017)	Intervention (ACR)	BESA (source montage)<br>sLORETA	Loudness (VAS)	Post- Vs. Pre-treatment	↓ loudness<br>↑ PAC (α) ↓ PAC (β,γ,δ,θ)
Balkenhol et al. (2013)	RS EEG	none (mean power over all electrodes)	Loudness (Matching)<br>Distress (TQ)<br>Hearing loss (PTA)	Correlation<br>Correlation<br>Correlation	↑ γ<br>↑ δ,θ<br>↓ γ
De Ridder and Vanneste (2014)	Intervention (EDS over AC)	sLORETA<br>LPC	Loudness (VAS)	Responders (Vs. Non-Responders)	↑ l pHC (β) ↑ HC (β) ↑ AMY (β) ↑ l INS (β) ↑ pHC (γ) ↑FPC (γ)<br>↑ l 2AC ↔ r pHC (δ) ↑ l 2AC ↔ r HC ↔ l pHC (θ) ↑ r PAC ↔ r pHC (β)<br>↑ pHC ↔ r PAC ↔ 2AC (β) ↑ pHC ↔ r PAC ↔ l 2AC (β)
De Ridder et al. (2011)	RS EEG with ICA	sLORETA<br>LPC	Distress (TQ)	Correlation	↑ Comp4 (sgACC, r IFG) (α, β) ↑ sgACC ↔ pHC ↔ OFC ↔ IFG (α, β)
De Ridder et al. (2013)	Intervention (rTMS over r DLPFC)	sLORETA<br>LPC	Loudness (VAS)	Responders (Vs. Non-Responders)	↑ r DLPFC ↔ l pHC ↔ l PAC↔ l 2AC (θ) ↑ PAC ↔ ACC ↔ pHC (θ)<br>↑ ACC ↔ r pHC ↔ PAC ↔ 2AC (θ)
De Ridder et al. (2015)	RS EEG	sLORETA	Loudness (NRS)<br>Loudness (matching)	Correlation<br>Correlation	↑ l aINS (α) ↑ rACC (β) ↑ dACC (β) ↑ l pHC (γ) ↑ PAC (β,γ)<br>none
Joos et al. (2012)	RS EEG	sLORETA	Distress (NRS)<br>Depression (BDI)	Correlation<br>Correlation	↑ r FPC (α,β) ↑ r OFC (α,β) ↑ sgACC (β)<br>↑ l FPC (α) ↑ l OFC (α)
Kim et al. (2016)	Intervention (TRT)	sLORETA<br>LPC	Distress (THI)<br><br>Loudness (NRS)<br>Awareness (NRS)	Correlation (improvement)<br><br>Correlation (improvement)<br>Correlation (improvement)	↑ l MFG (θ) ↑ l rACC (θ) ↑ r DLPFC ↑ l INS (α) ↑ r DLPFC (α)<br>↑ l rACC (α) ↑ pgACC (α) ↑ l IFG (α)<br>↓ r AC (γ,δ) ↓ pHC (β,δ,γ)<br>↑ r rACC (θ) ↑ r DLPFC (θ) ↑ rACC (α) ↑ pgACC (α) ↑ r DLPFC (α)<br>↑ l OFC (γ) ↑ r MFG (γ)

Meyer et al. (2014)	RS EEG	None	PCA:		
			Comp *Distress* (TQ, PRISM)	Correlation	↑ upper β over frontal electrodes
			Comp *Presence* (Duration, Loudness (VAS))	Correlation	↑ δ ↑ α ↓ γ over temporal and l perisylvian electrodes
Meyer et al. (2017)	RS EEG	sLORETA	Distress (TQ)	Correlation	↑ IPL/SMG (β) ↑ r pINS (β) ↑ r PP (β) ↑ r STG (β) ↑ INS (β)
			PCA:		
			Comp *Distress* (THI, TQ, PRISM)	Correlation	↑ INS (β)
			Comp *Affective disorders, health and QOL* (BDI, BAI, SCL-K-9, SF-36, WHOQOL-BREF)	Correlation	none
Pierzycki et al. (2015)	RS EEG	None (mean power over all electrodes)	Distress (THI, TFI, THQ)	Correlation	none
			QOL (WHOQOL-BREF)	Correlation	none
			Loudness (VAS)	Correlation	none
			PCA:		
			Comp *Tinnitus severity* (TFI, expect auditory subscale)	Correlation	none
			Comp *QOL* (WHOQOL-BREF, TFI- auditory subscale)	Correlation	none
			Comp *Hearing* (Duration, PTA, THQ- tinnitus and hearing subscale, TFI- auditory subscale)	Correlation	none
Schlee et al. (2014)	RS MEG	None	Duration	Correlation	↓ α variability over temporal sensors
Song et al. (2013a)	RS EEG	sLORETA<br>LPC	Age of Onset	Late (~ 52y) Vs. early onset (~ 29y)	↑ r OFC (γ) ↑ l DLPFC (β) ↑ r SMA (β) ↑ r SFG (β) ↑ r dACC (β)<br>↓ PCC (δ) ↓ r dPMC (θ)<br>↑ PAC ↔ 2AC (θ) ↑ l INS ↔ r INS (α) ↑ l INS ↔ r sgACC (α)<br>↑ r 2AC ↔ l PrC ↔ r PrC (α)
Song et al. (2013b)	Intervention (CI)	sLORETA<br>LPC	Loudness (NRS)	Slight (Vs. marked) improvement	↑ l 2AC (δ,γ) ↑ l TP (β) ↑ l PAC ↔ r PCC (δ)
			Distress (TQ)	Slight (Vs. marked) improvement	↑ l PAC ↔ r PAC (γ) ↑ r PAC ↔ l pHC (γ) ↑ r OFC ↔ l PrC (γ)
Song et al. (2013c)	RS EEG	sLORETA<br>LPC	Age of onset	Late (~ 52y) Vs. early onset (~ 29y)	↑ dACC (β) ↑ sgACC (β) ↑ pHC(β) ↑ r pgACC (γ) ↑ DLPFC (γ)<br>↑ r sgACC ↔ l PAC (γ) ↑ r MTG ↔ PAC (γ) ↑ r PCC ↔ PrC (γ)<br>↓ r PAC ↔ r PCC ↔ PrC (α) ↓ r PAC ↔ l PrC (β)
			Distress (TQ)	High (TQ: 47-84) Vs. low distress (TQ: 0-46)	↑ dACC (β) ↑ pgACC (γ) for late-onset<br>↑ l OFC (β,γ,δ) ↑ l SMG (α) ↑ l DLPFC (γ) for early-onset
Song et al. (2014)	RS EEG	sLORETA<br>LPC	Hyperacusis (HQ)	With Hyperacusis (Vs. without)	↑ SMA (β) ↑ dPMC (β) ↑ dACC (β) ↑ OFC (β) ↑ r AC (α)
					↑ r 2AC ↔ r PAC ↔ r PFC ↔ l sgACC ↑ l PAC ↔ l PCC
				Correlation	↑ OFC (β) ↑ r AC (α) ↑ dACC (β)

Song et al. (2015)	RS EEG	sLORETA<br>LPC	Awareness (%)	Correlation	↓ l dACC (δ) ↓ l pgACC (β,δ) ↓ pgACC (θ) ↓ rACC (β,δ,θ) ↓ sgACC (θ)<br>↓ l PAC ↔ rACC (β) ↓ l PAC ↔ sgACC (β)
Tass et al. (2012)	Intervention (ACR)	BESA (source montage)<br>sLORETA	Loudness (VAS)<br>Distress (TQ)	Post- Vs. Pre-treatment	↓ loudness, distress<br>↑ PAC (α) ↓ PAC (β,γ,δ,θ)
van der Loo et al. (2009)	RS EEG	LORETA	Loudness (VAS)	Correlation	↑ contralateral PAC (γ)
van der Loo et al. (2011)	RS EEG	sLORETA<br>LPC	Distress (TQ)	Correlation	↑ l aINS (α), r aINS (γ,δ)<br>↓ l aINS (γ,θ)
Vanneste and De Ridder (2011)	Intervention (tDCS over DLPFC)	sLORETA<br>LPC	Distress (VAS)<br>Loudness (VAS)	Post- Vs. Pre-treatment	↓ loudness, distress<br>↑ pgACC (α) ↓ r PAC (β,γ), iPSC (β,γ)<br>↑ r DLPFC ↔ pHC (θ) ↑ r PAC ↔ l pHC ↔ DLPFC ↔ pgACC (θ)<br>↓ DLPFC ↔ pgACC ↔ r PAC ↔ pHC (γ)<br>↓ l DLPFC ↔ l pHC ↔ pgACC (γ) ↓ r PAC ↔ r pHC ↔ pgACC (γ)
Vanneste and De Ridder (2012)	Intervention (alcohol)	sLORETA	Loudness (VAS)<br>Distress (VAS)	Post- Vs. Pre-treatment	↓ distress, loudness<br>↑ PCC (α) ↑ pgACC (β) ↑ dACC (β) ↑ l INS (β)<br>↓ OFC (α) ↓ VLPFC (α) ↓scACC (α) ↓ PrC (β) ↓ PrC (γ) ↓ PCC (γ)
Vanneste and De Ridder (2013)	RS EEG	sLORETA	Distress (TQ)	Correlation	↑ pgACC (α) ↑ sgACC (α)
Vanneste and De Ridder (2015)	RS EEG	sLORETA<br>LPC	Loudness (NRS)<br>Distress (TQ)	Correlation<br>Correlation	↑ AC (β,γ)<br>↑ sgACC (α,β) ↑ dACC (α,β), ↑PCC (α,β)<br>↑ PAC ↔ sgACC ↔ d ACC ↔ PCC (α,β)
Vanneste and De Ridder (2016)	RS EEG	sLORETA<br>LPC<br>Granger causality	Hearing loss (PTA)	Low hearing loss Vs. Controls	↑ l aMTG(θ); ↑ l PAC ↔ r PAC (γ)
				High hearing loss Vs. Controls	↑ pHC (θ); ↑ l PAC ↔ r PAC (α,θ) ↑ l pHC ↔ r pHC (α,θ);<br>↑ l pHC → l PAC (θ)
				High Vs. low hearing loss	↓ l aMTG (γ); ↑ l PAC ↔ r PAC ↑ l pHC ↔ r pHC (α,θ)<br>↓ l pHC → l PAC (θ)
			Mean hearing loss	Correlation	↑ pHC (θ) ↑ r pHC (α); ↑ l pHC ↔ r pHC ↔ l PAC (α)<br>↑ l pHC ↔ l PAC ↔ r PAC (θ) ↑ r pHC ↔ l PAC (θ); ↑ l pHC → l PAC (θ)
			Range of hearing loss	Correlation	↑ pHC (α,θ); ↑ l pHC ↔ l PAC ↔ r PAC (α,θ)<br>↑ l pHC ↔ r pHC ↔ r PAC (α); ↑ l pHC → l PAC (θ)
			Hearing loss at tinnitus frequency	Correlation	↑ l pHC ↔ l PAC ↔ r PAC (θ) ↑r pHC ↔ l PAC ↔ r PAC (θ)<br>↑ l pHC → l PAC (θ)

Vanneste et al. (2010a)	RS EEG	LORETA	Distress (TQ)	High Vs. low distress<br><br>High distress Vs. controls	↑ scACC (α) ↑ INS (α) ↑ pHC (α) ↑ AMY (α)<br>↓ PCC (α) ↓ PrC (α) ↓ DLPFC (α)<br>↑ dACC (α,β) ↓ dACC (δ,θ)
Vanneste et al. (2010b)	RS EEG	sLORETA	Type	Narrow-band noise (Vs. pure tone) tinnitus	↑ PCC (β) ↑ r HC (β) ↑ r pHC (γ)<br>↓ r lFPC (δ)
Vanneste et al. (2011a)	Intervention (tDCS over DLPFC)	sLORETA<br>LPC	Distress (VAS)<br>Loudness (VAS)	Responders (Vs. Non-Responders)	↑ r PAC (γ) ↑ r 2AC (γ) ↑ pHC (γ)<br>↑ r DLPFC ↔ r pHC (γ) ↑ r DLPFC ↔ sgACC (γ)
Vanneste et al. (2011b)	RS EEG	sLORETA	Location	Unilateral (Vs. bilateral) tinnitus<br>Bilateral (Vs. Controls)<br>Unilateral (Vs. Controls)	↑ VLPFC (δ) ↑ pHC (β,γ) ↑ AG (β,γ) ↑ AC (β,γ) ↓ sPMC (β)<br>↑ VLPFC (β) ↑ FPC (β) ↑ sPMC (γ)<br>↑ r sPMC(γ)
Vanneste et al. (2011c)	RS EEG	sLORETA	Location	Left- and right-sided tinnitus	↑ contralateral pHC (γ)
Vanneste et al. (2011d)	RS EEG	sLORETA<br>LPC	Duration	Recent onset (Vs. chronic = > 4 years) tinnitus	↑ SMA (θ) ↑ dACC (β) ↑ INS (β) ↑ PAC (γ) ↑ 2AC (γ)<br>↑ l pHC ↔ l PAC ↔ l 2AC ↔ l INS ↔ r DLPFC (γ)<br>↓ connectivity in general (α,γ,θ)
Vanneste et al. (2012)	RS EEG	sLORETA<br>LPC	Gender	Females (Vs. Males)	↑ OFC (β) ↑ FPC (β)<br>↑ OFC ↔ INS ↔ sgACC ↔ pHC ↔ PAC ↔ 2AC (α)
Vanneste et al. (2013)	Intervention (music)	sLORETA	Depression (HADS)<br>Loudness (VAS)<br>Annoyance (VAS)	Post- Vs. Pre-treatment (group with music to overcompensate hearing loss)	↑ loudness, annoyance, depressive feelings<br>↑ l dACC (α) ↑ l pgACC (β) ↑ PAC (γ)
Vanneste et al. (2014a)	RS EEG with ICA	sLORETA<br>LPC	Distress (TQ)<br><br><br><br>Loudness (VAS)	Correlation<br><br><br><br>Correlation	↓ Comp1 (PCC, PrC) (α,β) ↓ Comp2 (PCC, PrC, IPL, pHC) (α,β,γ)<br>↑ Comp4 (pgACC, sgACC, VMPFC, INS) (α)<br>↑ Comp6 (dACC, SMA, sgACC, VMPFC, MFG) (β)<br>Comp1 ↔ Comp2 ↔ Comp4 ↔ Comp6 (α,δ,θ)<br>↓ Comp3 (rsPCC, LG, pHC) (β)<br>↑ Comp5 (sgACC, VMPFC, HC, AMY, MFG) (β)<br>Comp3 ↔ Comp5 (γ)
Vanneste et al. (2014b)	RS EEG	sLORETA<br>LPC	Coping style<br>Distress (TQ)<br>Loudness (VAS)<br>Depression (BDI)	Maladaptive coping (Vs. adaptive coping)<br>Correlations	↑ loudness, distress, depression<br>↑ l DLPFC (α) ↑ sgACC (α); ↑ connectivity in default mode network<br>↑ DLPFC (α) for maladaptive coping<br>↑ sgACC (α) for distress and depression
Vanneste et al. (2016)	RS EEG	sLORETA	Cognition	Correlation	↑ HC (β) ↑ pgACC (β) ↑ sgACC (β) ↑ r INS (β)

Note: ↑, increase / positive correlation; ↓, decrease / negative correlation; ↔, functional connectivity between x and y; →, effective connectivity from x to y; l, left; r, right; 2AC, secondary auditory cortex; AC, auditory cortex; ACC, anterior cingulate cortex; ACR, acoustic coordinated reset; AG, angular gyrus; aINS, anterior insula; aMTG, anterior middle temporal gyrus; AMY, amygdala; BAI, Beck's Anxiety Inventory (Beck et al., 1988); BDI, Beck's Depression Inventory (Beck et al., 1961); CFC, cross-frequency coupling; CI, cochlear implantation; Comp, Component; dACC, dorsal anterior cingulate cortex; DLPFC, dorsolateral prefrontal cortex; dPMC, dorsal premotor cortex; EDS, extradural stimulation; FPC, frontopolar cortex; HC, hippocampus; ICA, independent component analysis; IFG, inferior frontal gyrus; INS, insula; IPL, inferior parietal lobule; iPSC, inferior primary somatosensory cortex; lFPC, lateral frontopolar cortex; LG, lingual gyrus; LPC, lagged phase coherence; MFG, middle frontal gyrus; MTG, middle temporal gyrus; NRS, numeric rating scale; OFC, orbitofrontal cortex; PAC, primary auditory cortex; PCA, principal component analysis; PCC, posterior cingulate cortex; PFC, prefrontal cortex; pgACC, pregenual anterior cingulate cortex; pHC, parahippocampus; pINS, posterior insula; PMC, premotor cortex; PP, planum parietale; PrC, precuneus; PRISM, Pictorial Representation of Illness and Self-Measure (Büchi et al., 1998); PTA, pure tone audiometry; QOL, quality of life; rACC, rostral anterior cingulate cortex; RS, resting-state; rsPCC, retrosplenial posterior cingulate cortex; rTMS, repetitive transcranial magnetic stimulation; scACC, subcallosal anterior cingulate cortex; SCL-K-9, Symptom Check List short form (Klaghofer and Brahler, 2001); SF-36, Short Form Health Survey (Ware Jr and Sherbourne, 1992); SFG, superior frontal gyrus; sgACC, subgenual anterior cingulate cortex; SMA, supplementary motor area; SMG, supramarginal gyrus; sPMC, superior premotor cortex; STG, superior temporal gyrus; TC, temporal cortex; tDCS, transcranial direct-current stimulation; TF, Tinnitus Functional Index (Meikle et al., 2012); THI, Tinnitus Handicap Questionnaire (Newman et al., 1996); THQ, Tinnitus Handicap Questionnaire (Kuk et al., 1990); TP, temporal pole; TQ, Tinnitus Questionnaire (Goebel and Hiller, 1994); TRT, tinnitus retraining therapy; VAS, visual analogue scale; VMPFC, ventromedial prefrontal cortex; WHOQOL-BREF, World Health Organization Quality of Life assessment (short form).

References

Adamchic, I., Langguth, B., Hauptmann, C., and Tass, P. A. (2014a). Abnormal cross-frequency coupling in the tinnitus network. Frontiers in neuroscience 8. doi: 10.3389/fnins.2014.00284.

Adamchic, I., Toth, T., Hauptmann, C., and Tass, P. A. (2014b). Reversing pathologically increased EEG power by acoustic coordinated reset neuromodulation. Hum. Brain Mapp. 35, 2099–2118. doi: 10.1002/hbm.22314.

Adamchic, I., Toth, T., Hauptmann, C., Walger, M., Langguth, B., Klingmann, I., and Tass, P. A. (2017). Acute effects and after-effects of acoustic coordinated reset neuromodulation in patients with chronic subjective tinnitus. NeuroImage. Clinical 15, 541–558. doi: 10.1016/j.nicl.2017.05.017.

Balkenhol, T., Wallhäusser-Franke, E., and Delb, W. (2013). Psychoacoustic tinnitus loudness and tinnitus-related distress show different associations with oscillatory brain activity. PLoS ONE 8, e53180. doi: 10.1371/journal.pone.0053180.

Beck, A. T., Epstein, N., Brown, G., and Steer, R. A. (1988). An inventory for measuring clinical anxiety. Psychometric properties. Journal of Consulting and Clinical Psychology 56, 893–897.

Beck, A. T., Ward, C. H., Mendelson, M., Mock, J., and Erbaugh, J. (1961). An inventory for measuring depression. Archives of general psychiatry 4, 561–571.

Büchi, S., Sensky, T., Sharpe, L., and Timberlake, N. (1998). Graphic representation of illness. A novel method of measuring patients' perceptions of the impact of illness. Psychotherapy and psychosomatics 67, 222–225.

De Ridder, D., Congedo, M., and Vanneste, S. (2015). The neural correlates of subjectively perceived and passively matched loudness perception in auditory phantom perception. Brain and behavior 5, e00331. doi: 10.1002/brb3.331.

De Ridder, D., Song, J.-J., and Vanneste, S. (2013). Frontal cortex TMS for tinnitus. Brain Stimulation 6, 355–362. doi: 10.1016/j.brs.2012.07.002.

De Ridder, D., and Vanneste, S. (2014). Targeting the Parahippocampal Area by Auditory Cortex Stimulation in Tinnitus. Brain Stimulation 7, 709–717. doi: 10.1016/j.brs.2014.04.004.

De Ridder, D., Vanneste, S., Congedo, M., and Koenig, T. (2011). The Distressed Brain. A Group Blind Source Separation Analysis on Tinnitus. PLoS ONE 6, e24273. doi: 10.1371/journal.pone.0024273.

Goebel, G., and Hiller, W. (1994). Tinnitus-Fragebogen (TF). Standardinstrument zur Graduierung des Tinnitusschweregrades. Ergebnisse einer Multicenterstudie mit dem Tinnitus-Fragebogen (TF) [The tinnitus questionnaire. A standard instrument for grading the degree of tinnitus. Results of a multicenter study with the tinnitus questionnaire]. HNO 42, 166–172.

Joos, K., Vanneste, S., and De Ridder, D. (2012). Disentangling Depression and Distress Networks in the Tinnitus Brain. PLoS ONE 7, e40544. doi: 10.1371/journal.pone.0040544.g001.

Kim, S. H., Jang, J. H., Lee, S.-Y., Han, J. J., Koo, J.-W., Vanneste, S., De Ridder, D., and Song, J.-J. (2016). Neural substrates predicting short-term improvement of tinnitus loudness and distress after modified tinnitus retraining therapy. Scientific reports 6, 29140. doi: 10.1038/srep29140.

Klaghofer, R., and Brahler, E. (2001). Construction and statistical testing of a short version of the SCL-90-R. Zeitschrift Für Klinische Psychologie Psychiatrie Und Psychotherapie 49, 115–124.

Kuk, F. K., Tyler, R. S., Russell, D., and Jordan, H. (1990). The psychometric properties of a tinnitus handicap questionnaire. Ear and hearing 11, 434–445.

Meikle, M. B., Henry, J. A., Griest, S. E., Stewart, B. J., Abrams, H. B., McArdle, R., Myers, P. J. et al. (2012). The tinnitus functional index. Development of a new clinical measure for chronic, intrusive tinnitus. Ear and hearing 33, 153–176. doi: 10.1097/AUD.0b013e31822f67c0.

Meyer, M., Luethi, M. S., Neff, P., Langer, N., and Büchi, S. (2014). Disentangling Tinnitus Distress and Tinnitus Presence by Means of EEG Power Analysis. Neural Plast. 2014, 1–13. doi: 10.1155/2014/468546.

Meyer, M., Neff, P., Grest, A., Hemsley, C., Weidt, S., and Kleinjung, T. (2017). EEG oscillatory power dissociates between distress- and depression-related psychopathology in subjective tinnitus. Brain Research 1663, 194–204. doi: 10.1016/j.brainres.2017.03.007.

Newman, C. W., Jacobson, G. P., and Spitzer, J. B. (1996). Development of the Tinnitus Handicap Inventory. Archives of otolaryngology--head & neck surgery 122, 143–148.

Pierzycki, R. H., McNamara, A. J., Hoare, D. J., and Hall, D. A. (2015). Whole scalp resting state EEG of oscillatory brain activity shows no parametric relationship with psychoacoustic and psychosocial assessment of tinnitus: A repeated measures study. Hear. Res. 331, 101–108. doi: 10.1016/j.heares.2015.11.003.

Schlee, W., Schecklmann, M., Lehner, A., Kreuzer, P. M., Vielsmeier, V., Poeppl, T. B., and Langguth, B. (2014). Reduced variability of auditory alpha activity in chronic tinnitus. Neural Plast. 2014, 436146. doi: 10.1155/2014/436146.

Song, J.-J., De Ridder, D., Schlee, W., van de Heyning, P., and Vanneste, S. (2013a). “Distressed aging”: the differences in brain activity between early- and late-onset tinnitus. Neurobiology of Aging 34, 1853–1863. doi: 10.1016/j.neurobiolaging.2013.01.014.

Song, J.-J., De Ridder, D., Weisz, N., Schlee, W., van de Heyning, P., and Vanneste, S. (2014). Hyperacusis-associated pathological resting-state brain oscillations in the tinnitus brain: a hyperresponsiveness network with paradoxically inactive auditory cortex. Brain Struct Funct 219, 1113–1128. doi: 10.1007/s00429-013-0555-1.

Song, J.-J., Punte, A. K., De Ridder, D., Vanneste, S., and van de Heyning, P. (2013b). Neural substrates predicting improvement of tinnitus after cochlear implantation in patients with single-sided deafness. Hear. Res. 299, 1–9. doi: 10.1016/j.heares.2013.02.001.

Song, J.-J., Vanneste, S., and De Ridder, D. (2015). Dysfunctional noise cancelling of the rostral anterior cingulate cortex in tinnitus patients. PLoS ONE 10, e0123538. doi: 10.1371/journal.pone.0123538.

Song, J.-J., Vanneste, S., Schlee, W., van de Heyning, P., and De Ridder, D. (2013c). Onset-related differences in neural substrates of tinnitus-related distress: the anterior cingulate cortex in late-onset tinnitus, and the frontal cortex in early-onset tinnitus. Brain Struct Funct. doi: 10.1007/s00429-013-0648-x.

Tass, P. A., Adamchic, I., Freund, H.-J., Stackelber, T. von, and Hauptmann, C. (2012). Counteracting tinnitus by acoustic coordinated reset neuromodulation. Restor. Neurol. Neurosci. 30, 137–159.

van der Loo, E., Congedo, M., Vanneste, S., van Heyning, P. de, and de Ridder, D. (2011). Insular lateralization in tinnitus distress. Autonomic Neuroscience 165, 191–194. doi: 10.1016/j.autneu.2011.06.007.

van der Loo, E., Gais, S., Congedo, M., Vanneste, S., Plazier, M., Menovsky, T., van de Heyning, P., De Ridder, D., and Greenlee, M. W. (2009). Tinnitus Intensity Dependent Gamma Oscillations of the Contralateral Auditory Cortex. PLoS ONE 4, e7396. doi: 10.1371/journal.pone.0007396.

Vanneste, S., Congedo, M., and de Ridder, D. (2014a). Pinpointing a Highly Specific Pathological Functional Connection That Turns Phantom Sound into Distress. Cerebral Cortex 24, 2268–2282. doi: 10.1093/cercor/bht068.

Vanneste, S., and De Ridder, D. (2011). Bifrontal transcranial direct current stimulation modulates tinnitus intensity and tinnitus-distress-related brain activity. The European journal of neuroscience 34, 605–614. doi: 10.1111/j.1460-9568.2011.07778.x.

Vanneste, S., and De Ridder, D. (2012). The use of alcohol as a moderator for tinnitus-related distress. Brain Topogr 25, 97–105. doi: 10.1007/s10548-011-0191-0.

Vanneste, S., and De Ridder, D. (2013). Brain areas controlling heart rate variability in tinnitus and tinnitus-related distress. PLoS ONE 8, e59728. doi: 10.1371/journal.pone.0059728.

Vanneste, S., and De Ridder, D. (2015). Stress-Related Functional Connectivity Changes Between Auditory Cortex and Cingulate in Tinnitus. Brain connectivity 5, 371–383. doi: 10.1089/brain.2014.0255.

Vanneste, S., and De Ridder, D. (2016). Deafferentation-based pathophysiological differences in phantom sound: Tinnitus with and without hearing loss. Neuroimage. doi: 10.1016/j.neuroimage.2015.12.002.

Vanneste, S., Faber, M., Langguth, B., and De Ridder, D. (2016). The neural correlates of cognitive dysfunction in phantom sounds. Brain Research. doi: 10.1016/j.brainres.2016.03.016.

Vanneste, S., Focquaert, F., van de Heyning, P., and De Ridder, D. (2011a). Different resting state brain activity and functional connectivity in patients who respond and not respond to bifrontal tDCS for tinnitus suppression. Experimental brain research 210, 217–227. doi: 10.1007/s00221-011-2617-z.

Vanneste, S., Joos, K., and De Ridder, D. (2012). Prefrontal cortex based sex differences in tinnitus perception. Same tinnitus intensity, same tinnitus distress, different mood. PLoS ONE 7, e31182. doi: 10.1371/journal.pone.0031182.

Vanneste, S., Joos, K., Langguth, B., To, W. T., and De Ridder, D. (2014b). Neuronal correlates of maladaptive coping. An EEG-study in tinnitus patients. PLoS ONE 9, e88253. doi: 10.1371/journal.pone.0088253.

Vanneste, S., Plazier, M., van der Loo, E., van de Heyning, P., Congedo, M., and De Ridder, D. (2010a). The neural correlates of tinnitus-related distress. Neuroimage 52, 470–480.

Vanneste, S., Plazier, M., van der Loo, E., van de Heyning, P., and De Ridder, D. (2010b). The differences in brain activity between narrow band noise and pure tone tinnitus. PLoS ONE 5, e13618. doi: 10.1371/journal.pone.0013618.

Vanneste, S., Plazier, M., van der Loo, E., van de Heyning, P., and De Ridder, D. (2011b). The difference between uni- and bilateral auditory phantom percept. Clinical neurophysiology : official journal of the International Federation of Clinical Neurophysiology 122, 578–587. doi: 10.1016/j.clinph.2010.07.022.

Vanneste, S., van de Heyning, P., and de Ridder, D. (2011c). Contralateral parahippocampal gamma-band activity determines noise-like tinnitus laterality: a region of interest analysis. Neuroscience 199, 481–490. doi: 10.1016/j.neuroscience.2011.07.067.

Vanneste, S., van de Heyning, P., and De Ridder, D. (2011d). The neural network of phantom sound changes over time. A comparison between recent-onset and chronic tinnitus patients. European Journal of Neuroscience 34, 718–731. doi: 10.1111/j.1460-9568.2011.07793.x.

Vanneste, S., van Dongen, M., Vree, B. de, Hiseni, S., van der Velden, E., Strydis, C., Joos, K., Noreña, A. J., Serdijn, W., and De Ridder, D. (2013). Does enriched acoustic environment in humans abolish chronic tinnitus clinically and electrophysiologically? A double blind placebo controlled study. Hear. Res. 296, 141–148. doi: 10.1016/j.heares.2012.10.003.

Ware Jr, J. E., and Sherbourne, C. D. (1992). The MOS 36-item short-form health survey. I. Conceptual framework and item selection. Medical care, 473–483.
